# The discovery of endogenous retroviruses

**DOI:** 10.1186/1742-4690-3-67

**Published:** 2006-10-03

**Authors:** Robin A Weiss

**Affiliations:** 1 Division of Infection & Immunity, University College London, 46 Cleveland Street, London W1T 4JF, UK

## Abstract

When endogenous retroviruses (ERV) were discovered in the late 1960s, the Mendelian inheritance of retroviral genomes by their hosts was an entirely new concept. Indeed Howard M Temin's DNA provirus hypothesis enunciated in 1964 was not generally accepted, and reverse transcriptase was yet to be discovered. Nonetheless, the evidence that we accrued in the pre-molecular era has stood the test of time, and our hypothesis on ERV, which one reviewer described as 'impossible', proved to be correct. Here I recount some of the key observations in birds and mammals that led to the discovery of ERV, and comment on their evolution, cross-species dispersion, and what remains to be elucidated.

## Background

If Charles Darwin reappeared today, he might be surprised to learn that humans are descended from viruses as well as from apes. Some 8% of human DNA represents fossil retroviral genomes, and that is not counting the LINE elements and other retrotransposons that are scattered so liberally across our genome [1,2]. Darwin might be reassured that we share most though not all of these insertions with chimpanzees [3,4]. But how did endogenous viruses first come to light?

The discovery of ERV took place in the late 1960s and early 1970s. Three types of ERV were found around the same time: avian leukosis virus in the domestic fowl (*Gallus gallus*), and murine leukemia virus and murine mammary tumor virus in the laboratory mouse (*Mus musculus*). Initially, ERV were discovered by combining virological and immunological methods with Mendelian genetics; their existence was then confirmed by nucleic acid hybridization.

**Figure 1:**
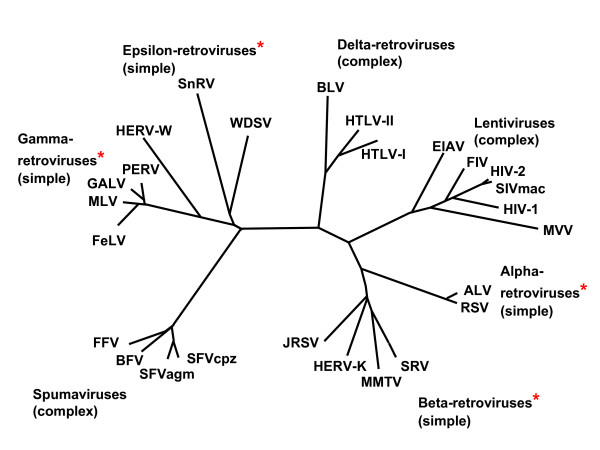
Phylogeny of Retroviruses: genera that include endogenous genomes are marked with an asterisk.

Retroviruses can be classified as those that have simple genomes – the alpha, beta, gamma and epsilon retroviruses, and those with complex genomes – the lentiviruses, deltaviruses and spumaviruses (Figure 1). Only the simple retroviruses have become endogenous in their hosts, with the questionable exception of spumaviruses. Why this should be so is not understood.

### Retroviruses and the provirus hypothesisis

Although retroviruses did not gain their name until 1974 [5], retroviral diseases were distinguished much earlier. Bovine leukosis and Jaagsiekte in sheep were recognized in the 19th century. In 1904, Vallée and Carré showed that equine anemia was infectiously transmitted by a filtrate and we now know that the etiologic agent is a lentivirus. Oncogenic retroviruses have been studied ever since erythroleukemia in chickens was shown to be experimentally transmissible in 1908 by Ellermann and Bang, and the transfer of sarcoma in chickens through filtrates by Rous in 1911 and by Fujinami and Inamoto in 1914 [6,7].

In 1961 Rous sarcoma virus (RSV) particles were shown to contain RNA [8] and thus oncogenic retroviruses were called RNA tumor viruses. However, cells transformed by RSV maintained stable properties through many mitoses. This heritability of virus-transformed phenotype, even in the absence of viral replication [9], led Howard Temin to postulate that in the infected cell, the RSV genome made a DNA copy which then integrated into host chromosomal DNA [10]. Temin called his concept the DNA provirus hypothesis by analogy with the integrated prophage of temperate bacteriophage. Indeed, André Lwoff, who won a Nobel Prize for discovering prophage and lysogeny, had suggested integration of the DNA tumor virus, polyoma virus [11]. Thus the concept of integration of DNA tumor virus genomes in transformed somatic cells was debated, and was demonstrated in 1968 [12]. However, the notion of Mendelian transmission of integrated genomes of RNA tumor viruses in the germ-line of healthy animals was regarded as bizarre.

Conversely, non-Mendelian inheritance of genetic markers was also puzzling geneticists at that time. For example, Barbara McClintock was studying "jumping genes" in maize, as she relates in her 1983 Nobel Prize address [13]. It was only much later that many of these strange transpositions in maize and Drosophila were found to be effected by retrotransposons.

### Endogenous avian leukosis viruses (ALV)

ALV is an alpha-retrovirus. Chickens infected *in ovo *frequently develop lymphoid leukosis, which is a B-cell leukemia arising from infected cells in the bursa of Fabricius. ALV replicates in chick embryo fibroblasts but does not transform them. Rous sarcoma virus (RSV) is closely related but carries the *src *oncogene and transforms fibroblasts. These viruses have a simple genome organisation:

ALV: 5' LTR-*gag*-*pol*-*env*-LTR 3'

RSV (Bryan): 5' LTR-*gag*-*pol*-*src*-LTR 3'

RSV (Prague): 5' LTR-*gag*-*pol*-*env*-*src*-LTR 3'

In America, the Bryan strain of RSV was chiefly studied, which is defective for replication because the *src *gene is substituted for the *env *gene. In Europe, non-defective RSV strains (Prague, Schmidt-Ruppin and Carr-Zilber) were studied, which carry *src *in addition to the replicative genes. Defective Bryan RSV can be rescued by ALV which supplies the missing Env glycoproteins. As a provider of this complementing Env, ALV was called a helper virus [14]. Different envelope 'subgroups' – or serotypes – of ALV are distinguished by neutralizing sera and by utilizing distinct cell surface receptors [15,16] and the RSV particles with ALV envelopes were named 'pseudotypes' [15].

In the 1960s, avian leukosis was becoming an increasing problem in egg-laying hens, and efforts were made to maintain leukosis-free flocks. To screen for leukosis, a serologic test was devized for 'group-specific' antigen, which was common to all ALV serotypes [17]. This was done by complement fixation (ELISA technology had not yet been invented), and it was called the COFAL test. We now know that group-specific antigen is the major capsid antigen (CA), p27. In fact, the term Gag was coined [5] as an acronym for group-specific antigen.

Robert Dougherty became concerned that the COFAL test was apparently not sufficiently specific because certain uninfected chickens gave positive results [18]. Later his team also detected virus-like particles as well as Gag-related antigen in 'ALV-free' chicken tissue [19]. Then Payne and Chubb [20] demonstrated that Gag-related antigen was inherited as a dominant Mendelian gene in crosses between Gag-positive and Gag-negative inbred lines of chicken. The question remained whether the endogenous antigen was encoded by a latent retroviral genome or whether it represented a normal host protein with a cross-reacting epitope.

I first heard Payne's preliminary results at the European Tumor Virus Workshop at Sorrento, Italy, in April 1967. I was enthralled because I was puzzling over a different problem as part of my doctoral studies. I had found that fibroblast cultures of some chick embryos but not others, allowed the release of infectious Bryan RSV in the apparent absence of a helper leukosis virus [21]. Peter K Vogt observed the same phenomenon and found that the virus infected Japanese quail cells [22]. I then found that the envelope of the 'helper-free' RSV was novel in its receptor specificity and neutralization properties [23,24]. Later, Hidesaburo Hanafusa's laboratory published similar data [25] and called the activity 'chick helper factor'. It thus became apparent that some normal chick cells could provide the missing Env protein to complement Bryan RSV.

When I first submitted my results in 1968 on a novel 'endogenous' envelope, suggesting the existence of an integrated retrovirus in normal embryo cells, the manuscript was roundly rejected; one reviewer pronounced that my interpretation was impossible! Clearly this reviewer had no time for Temin's provirus hypothesis either. Later that year, Howard Temin visited me in London because my short 1967 paper [21] had aroused his curiosity. He pored over my lab notebooks very critically, and after some 4 hours of intense discussion he urged me to try publishing it again. I was most grateful to him and to the Journal of General Virology when my work was finally accepted [23,24]. George Todaro also visited me in 1968 and cited my data in his and Huebner's hypothesis on latent retroviruses that first coined the term 'oncogene' [26].

Mendelian inheritance of a Gag-like antigen and complementation of an Env-defective strain of RSV comprised two separate lines of evidence that something related to a retrovirus existed in normal embryo cells. So the next step was to collaborate with Jim Payne to determine whether Env complementation and Gag expression were inherited concomitantly. Using inbred chickens, F1 hybrids and back-crosses, we found that both phenotypes were indeed inherited according to Mendel's first law and that they segregated together as a single locus [27]. A complete, infectious endogenous virus was not released in our birds although both Gag and Env were expressed, but we obtained evidence for release of infectious virus after treatment of cells with X-rays. Meanwhile, Vogt and Friis [28] had found that a different line of chickens spontaneously released infectious virus with identical envelope properties to the one we were studying.

After I joined Peter Vogt's laboratory in 1970, we were able to show that treatment of normal chicken cells with a variety of activating agents such as ionizing radiation or carcinogens stimulated release of virus [29]. Curiously, we found that both inbred lines of chicken, positive or negative for Gag and Env expression, produced virus after physical or chemical activation. It was later shown that the induced virus originated from a different provirus than that expressing Gag and Env [30].

When I came to Vogt's group, reverse transcriptase (RT) had recently been discovered [31,32] and we used RT activity to measure release of virus particles [29]. With Temin's provirus hypothesis vindicated by the discovery of RT, it seemed opportune to investigate whether normal, uninfected chickens contained proviral DNA. Using labelled ALV RNA, it was possible to detect related DNA sequences by Cot hybridization [33-35]. After Southern blotting techniques were developed, many proviral copies were found to be present in most chicken breeds [36]. Individual proviral loci were characterized and mapped [30]; many represent incomplete or defective genomes [37].

I was interested to know if the chicken ERV was a recent introduction into domestic fowl, or whether it was present in the ancestor species, the red jungle fowl. In 1970, I made a field trip to Malaysia and lived with tribesmen (orang asli) in the Pahang jungle who knew how to trap these birds, in order to take blood samples and to collect eggs for cell culture. The red jungle fowl carried endogenous ALV [38]. We later found that the three other extant species of the same genus, *Gallus*, did not possess endogenous ALV [39]. Apparently this ERV colonized the chicken germ-line after speciation but before domestication.

**Figure 2:**
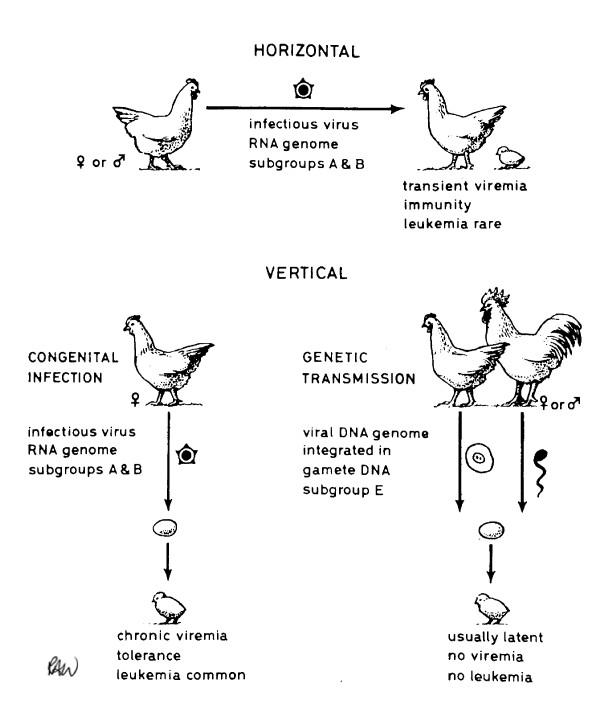
Exogenous and endogenous modes of transmission of ALV.

The modes of transmission of exogenous and endogenous ALV are shown in Figure 2. However, the situation is more complex than that depicted because exogenous infection leads to the generation of recombinant viruses at high frequency, provided that endogenous *env *sequences are expressed [40]. We therefore postulated that genetic exchange occurs through mixed assembly of RNA genomes in virus particles, followed by molecular recombination upon reverse transcription in the next replicative cycle. A similar recombination phenomenon with endogenous *env *transcripts of gamma-retroviruses in mice and cats is part of the pathway of leukemogenesis. Expression of endogenous Env can also block receptors on chicken cells to incoming virus [41] so that the endogenous virus has a potentially xenotropic host range, an effect equivalent to the Fv-4 endogenous viral gene described later in mice.

Astrin et al [42] identified a rooster that lacked any integrated provirus and a line of chickens was eventually bred from this bird. The generation of birds without endogenous ALV sequences indicated that viral genomes were not essential for host functions. However, these chickens do carry a second family of ERV called endogenous avian virus (EAV) although they are not infectious. EAV sequences are present in DNA of all species of *Gallus *and therefore have a more ancient origin [43].

More recently, the characterization of a highly virulent strain of ALV (ALV-J) causing myeloid leukemia in broilers showed that it was a recombinant virus, with ALV *gag *and *pol *and an EAV-related *env *gene [44]. This is reminiscent of the chimeric genome of the endogenous genome in cats derived from baboons (discussed later) which is a recombinant between a gamma-retrovirus related to murine leukemia virus and a beta-retrovirus related to Mason-Pfizer monkey virus [45]. The cellular receptor for the ALV-J virus has recently been identified [46].

A third group of avian retroviruses includes the reticulo-endotheliosis virus (REV) of turkeys, which probably had a mammalian origin. Interestingly REV has not integrated into germ line DNA but both REV and ALV have inserted into the circular DNA of Marek's disease herpesvirus [47] and REV has also integrated into fowlpox genomes [48,49]. Thus retroviruses have become 'endogenous' in the genome of larger, more complex DNA viruses.

### Murine leukemia virus (MLV) and mammalian gamma-retroviruses

Thymic lymphomagenesis in mice follows activation of endogenous MLV but this was not appreciated until 1970 [50]. In 1933 Jacob Furth bred the AKR mouse strain that has a high probability of developing lymphoma, but MLV was not discovered as a virus until 1951, by Ludwig Gross [7]. AKR mice, carrying two endogenous genomes of N-tropic MLV, can replicate activated virus as they carry a permissive allele of the *Fv-1 *cellular restriction gene [51]. They begin to release virus spontaneously as late embryos [50].

Spontaneous release of MLV from uninfected murine cell cultures was observed by Aaronson et al [52]. At the same time as we found we could induce ERV production in chick embryo cells [29] similar experiments were reported for MLV activation by halogenated pyrimidines [53,54]. In fact, radiation-induced lymphomagenesis with virus activation had been reported in mice earlier [55,56]. At that time, however, *in vivo *activation of a latent exogenous virus could not be distinguished from an endogenous genome in the germ-line. The genetic mapping and analysis of viral gene expression of endogenous MLV was studied in great detail in the 1970s and 1980s [37]. As with endogenous ALV many of the genomes are defective, while others maintain open reading frames or complete, potentially infectious genomes.

The induction of thymic lymphomas in AKR and other susceptible mice involves more than activation of MLV. The AK virus in viremic mice recombines with other endogenous *env *genes, and it is these recombinant retroviruses with expanded tropism that elicit malignancy following integration adjacent to proto-oncogenes [57]. There is an analogous situation in cats except that the initiating feline leukemia virus subtype A is an exogenous infection which then forms lymphomagenic recombinants with endogenous *env*, giving rise to FeLV-B [57].

**Figure 3:**
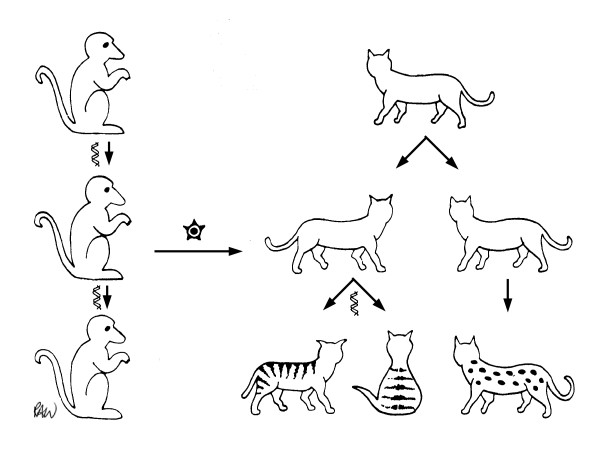
Exit from and entry into host genomes: transmission of the baboon ERV, BaEV to become the feline ERV, RD114.

With the discovery of endogenous MLV, many investigators in the early 1970s began to examine cells from other species for similar viruses. Reverse transcriptase assays, electron microscopy and nucleic acid hybridization provided useful methods of detection. Many mammalian species were found to harbor gamma-retroviruses related to MLV, including non-human primates. For instance gamma-retrovirus was isolated from trophoblastic cells of the baboon placenta [58]. This virus was found to be very closely related antigenically and by sequence homology to the endogenous RD114 virus in cats (which is itself unrelated to endogenous FeLV). Benveniste and Todaro [59] observed, like we did for jungle fowl, that only certain species of the cat genus, *Felis*, possessed this endogenous genome related to the baboon ERV. In contrast, all species of baboons [60] carry this virus so it would appear to have been present in the germ line of primates much longer than in cats. Thus it seems evident that a horizontal, infectious event occurred to transfer the virus from baboons to cats, whereupon it became endogenous in the new species (Figure 3).

Since cats would be quite likely to scavenge and feed on baboon placentae, a possible exposure to the virus can be envisioned. The human placenta is also permissive to the expression of multiple families of human endogenous retrovirus (HERV) genomes. Indeed, it appears that the retroviral envelope glycoproteins of at least one of them (HERV-W and possibly ERV-3) may be involved in natural syncytium induction to form the syncytiotrophoblast [61-63].

### Murine mammary tumor virus (MMTV)

Susceptibility to breast cancer in mice was initially thought to be genetic because high and low incidence strains of mice seemed to breed true. In 1936, however, J. J. Bittner showed that foster-nursing a low-incidence strain of new born mice on high-incidence mothers caused the females to develop breast cancer as adults [7]. Eventually, observations of a filterable oncogenic agent in the milk led to the identification of the MMTV in 1949 by L. Dmochowski in electronmicrographs. However, in 1952 both Bittner and Otto Mühlbock observed that in certain mouse strains, mammary tumor predisposition could be transmitted by the male. It was thought that virus was transmitted in the semen to the female, to infect fetuses in turn [7].

MMTV was discovered to be endogenous at the same time as endogenous ALV. During the 1967 conference at which Payne described Mendelian inheritance of Gag antigen and I reported Env complementation in chickens, a young investigator with Mühlbock at the Netherlands Cancer Institute, Peter Bentvelzen, reported that the inherited mammary cancer in GR mice was associated with MMTV production. By the time he published this study, Bentvelzen and colleagues had evidence to suggest that the virus itself was the inherited factor [64,65].

As with endogenous ALV and MLV, mice carry numerous MMTV ERV in their chromosomes [66]. Later, Acha-Orbea showed that these MMTV loci encode superantigens [67].

### Xenotropism and xenotransplantation

Many endogenous retroviruses do not readily re-infect their own host cells but can infect other species *in vitro *or *in vivo*. Thus the endogenous ALV of chickens infects cells of quail, pheasants and turkey more readily than the chicken [22,23]. Jay Levy studied New Zealand black mice with auto-immune disease and discovered an endogenous MLV strain that could infected human and rat cells but not murine cells. He coined the term 'xenotropic' for viruses that only infect foreign species [68] in contrast to 'ecotropic' and 'amphotropic' strains. Thus the reservoir of infection may be a DNA provirus in the chromosomes of one species while the virus produced from it may infect other species.

There is a selective advantage for the host to be insusceptible to re-infection by a potentially pathogenic ERV, because, when a few cells spontaneously release virus, it cannot then be amplified to reach a high viral load. Resistance mechanisms include mutation of receptors, blocking of receptors by endogenous Env expression, and intracellular restriction factors [51,69].

The feline ERV RD114 is an interesting example of xenotropism. It was first detected in the human rhabdomyosarcoma cell line, RD, and its discovery was hailed as the first human RNA tumor virus [70]. When several groups showed that RD114 virus was actually an endogenous cat virus, it was realized that the human RD cell line had been passaged as a xenograft in the brain of a fetal kitten – this was a convenient immunologically privileged site before immunodeficient mice were available. Human tumor xenografts in mice also become infected with xenotropic MLV [71]. There is recent evidence that a gammaretrovirus related to xenotropic MLV is present in a small proportion of patients with prostate cancer [72].

If human tumors can pick up retroviruses when xenografted into animals, it follows that cross-species infection might also occur if animal tissues were to be xenotransplanted into humans. That is why we investigated pig endogenous retroviruses (PERV) and found that two of three envelope subgroups could infect human cells *in vitro *[73]. Thankfully there is no evidence to date of PERV infection *in vivo *in exposed humans [74]. Murine hybridomas can also release xenotropic MLV, so it is important to ensure that biologic medicines such as therapeutic monoclonal antibodies are not contaminated by retroviruses [75].

### ERV and retroviral vectors

ERV expression can affect retroviral vectors in two ways. First, their transcripts can be packaged alongside the gene of choice and thus constitute contaminating genetic material in gene therapy formulations. Although the murine packaging cell lines do not express endogenous MLV genomes they do express VL30 ERV and other sequences which can represent 50% or more of the vector stock and which are transferred to primates [76]. Adoption of packaging lines of other species such as the dog will exclude VL30, but so little research of canine ERV has been done that the potential hazard remains unknown. Regarding human packaging cells, there is no evidence that HERVs are incorporated into MLV-based [77] or lentiviral vectors.

Second, ERV expression might mobilize genomes containing therapeutic genes if the packaging signals remain intact, and they might generate replication-competent recombinants. Since humans do not produce infectious HERV, mobilization appears unlikely, and MLV-based genomes are not cross-packaged into expressed HERV particles [77].

### Evolutionary perspective

Retroviral genomes and other retro-elements such as *Alu *and LINE sequences are widely dispersed among hosts [37]. Do such insertions simply represent "junk" DNA, or do they play a role in genetic regulation of the host? Do retroviruses serve as vectors for horizontal gene exchange? Do ERVs always become defective over time?

**Figure 4:**
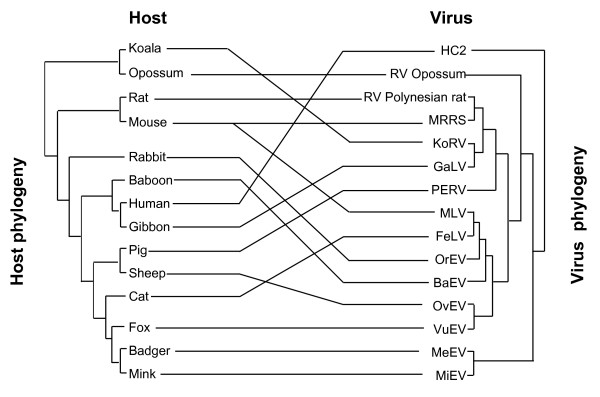
Co-evolution and cross-species infection of MLV-related genomes among mammals. Host and retroviral phylogenies are shown on the left and right respectively. Horizontal links indicate co-evolution, whereas sloping links show cross-species infection across large host taxa. Thus two closely related retroviruses infect an ape (gibbon) and a marsupial (koala), and two closely related ERV genomes are found in a carnivore (fox) and a ruminant (sheep). Adapted from Martin *et al*. [79].

MLV-related gamma-retroviruses may reside for millions of years in the germ-line in one group of animals, as we showed for old world pig species [78], and yet remain replication competent [73]. Maintenance of functional genomes with open reading frames probably requires retrotransposition and therefore complete genomes tend to be recently recycled ones. M. Tristem's group [79] has demonstrated multiple host switching of ERV (Figure 4). Colonization of a new host presumably goes via an infectious phase before insertions occur in its germ-line.

Different bursts of endogenization have occurred at different times. This has been exemplified for beta-retroviruses related to HERV-K in old world primates [2,3]. Such a process of endogenization currently appears to be taking place with a highly leukemogenic gamma-retrovirus of the Koala in Australia [80]. Endogenization may eventually help to modulate viral load and pathogenicity if it acts as a dominant negative factor to related exogenous viruses.

As Mendelian elements, retroviruses must be subject to host selection. However, with the exception of enrolling *env *genes in placental differentiation, ERV appear to be parasitic DNA sequences for which the host has little use, other than to protect against further retrovirus infection. Potentially, ERV can damage the host by mutational insertion and by homologous recombination. But despite a tendency to implicate ERV in many 'non-infectious' diseases in humans, there is scant evidence that they play a significant role [1]. There are only rare examples where a recessive single gene disorder in a family lineage is caused by an endogenous retroviral insertion disrupting gene function [2,3].

Given the propensity of retroviruses to switch between transmission as infectious agents and as host Mendelian elements, and given that they are able to transduce host genes to become viral oncogenes, it seems strange that there are no examples of gene transduction by retroviruses into the germline of new hosts. Retroviruses could in theory serve as a horizontal means of exchange of genetic information, like transducing lysogenic bacteriophage. However, other than transporting themselves, ERV do not appear to be purveyors of genes; even the retroviruses that bear oncogenes are not recorded as being naturally transmitted from host to host.

Finally, one may ask why DNA viruses that have a capacity to integrate into host DNA have not been detected in the germ line. Although integration is not an obligate step in their replication cycles, polyoma viruses, papilloma viruses, hepadnaviruses, adenoviruses and parvoviruses could each have gained a free ride to the next host generation, provided they were able to infect primordial germ cells or early embryo cells before segregation of the germ line. Adeno-associated virus has a preferred integration site on human chromosome 19 but has apparently not become inherited at this locus. Like MLV [81], the polyoma virus, SV40, can infect embryonal stem cells *in vitro*, and become latent in them [82]. This would be a good way to endogenize yet there is little evidence that it has happened. I am aware of only one example of a Mendelian DNA virus, that of human herpesvirus 6 [83,84], and this is not universal in the human population. It will be fascinating to work out why HHV-6 but not other herpesviruses endogenize, and whether other non-retroviral endogenous genomes will be discovered.

## Conclusion

ERV were discovered through the careful analysis of virological and immunological markers that appeared to be inherited by the host as Mendelian traits. Interestingly, the crucial evidence of endogenous ALV, MLV and MMTV came to light in the same period in the late 1960s. The discovery of reverse transcriptase in 1970 made these strange findings plausible. Later molecular genetic studies showed that the genomes of all vertebrate species studied have been colonized by multiple sets of retrovirus. Phylogenetic studies of viral genomes indicate that the introduction of ERV proceeds in waves with relatively rapid amplification of copy numbers and dispersal in the host genome. Their functions, if any, in the host remain an enigma, except for *env *genes driving differentiation of the syncytiotrophoblast in the placenta.

## Competing interests

The author(s) declare that they have no competing interests.

## References

[B1] Griffiths DJ (2001). Endogenous retroviruses in the human genome sequence. Genome Biol.

[B2] Villesen P, Aagaard L, Wiuf C, Pedersen FS (2004). Identification of endogenous retroviral reading frames in the human genome. Retrovirology.

[B3] Hughes JF, Coffin JM (2001). Evidence for genomic rearrangements mediated by human endogenous retroviruses during primate evolution. Nat Genet.

[B4] Barbulescu M, Turner G, Seaman MI, Deinard AS, Kidd KK, Lenz J (1999). Many human endogenous retrovirus K (HERV-K) proviruses are unique to humans. Curr Biol.

[B5] Baltimore D (1975). Tumor viruses: 1974.

[B6] Vogt PK, Coffin JM, Hughes SH and Varmus HE (1997). Historical introduction to the general properties of retroviruses. Retroviruses.

[B7] Gross L (1980). Oncogenic viruses.

[B8] Crawford LV, Crawford EM (1961). The properties of Rous sarcoma virus purified by density gradient centrifugation. Virology.

[B9] Temin HM (1963). Separation of morphological conversion and virus production in Rous sarcoma virus infection. Cold Spring Harbor Symp Quant Biol.

[B10] Temin HM (1964). Nature of the provirus of Rous sarcoma.. Nat Cancer Inst Monogr.

[B11] Lwoff A (1960). Tumor viruses and the cancer problem: a summation of the conference. Cancer Res.

[B12] Sambrook J, Westphal H, Srinivasan PR, Dulbecco R (1968). The integrated state of viral DNA in SV40-transformed cells. Proc Natl Acad Sci U S A.

[B13] McClintock B (1984). The significance of responses of the genome to challenge. Science.

[B14] Hanafusa H, Hanafusa T, Rubin H (1963). The defectiveness of Rous sarcoma virus. Proc Natl Acad Sci U S A.

[B15] Rubin H (1965). Genetic control of cellular susceptibility to pseudotypes of Rous sarcoma virus. Virology.

[B16] Vogt PK, Ishizaki R (1965). Reciprocal patterns of genetic resistance to avian tumor viruses in two lines of chickens. Virology.

[B17] Sarma PS, Turner HC, Huebner RJ (1964). An avian leucosis group-specific complement fixation reaction. Application for the detection and assay of non-cytopathogenic leucosis viruses. Virology.

[B18] Dougherty RM, Di Stefano HS (1966). Lack of relationship between infection with avian leukosis virus and the presence of COFAL antigen in chick embryos. Virology.

[B19] Dougherty RM, Di Stefano HS, Roth FK (1967). Virus particles and viral antigens in chicken tissues free of infectious avian leukosis virus. Proc Natl Acad Sci U S A.

[B20] Payne LN, Chubb RC (1968). Studies on the nature and genetic control of an antigen in normal chick embryos which reacts in the COFAL test. J Gen Virol.

[B21] Weiss RA (1967). Spontaneous virus production from 'non-virus producing' Rous sarcoma cells. Virology.

[B22] Vogt PK (1967). A virus released by "nonproducing" Rous sarcoma cells. Proc Natl Acad Sci U S A.

[B23] Weiss RA (1969). The host range of Bryan strain Rous sarcoma virus synthesized in the absence of helper virus.. J Gen Virol.

[B24] Weiss RA (1969). Interference and neutralization studies with Bryan strain Rous sarcoma virus synthesized in the absence of helper virus. J Gen Virol.

[B25] Hanafusa H, Miyamoto T, Hanafusa T (1970). A cell-associated factor essential for formation of an infectious form of Rous sarcoma virus. Proc Natl Acad Sci U S A.

[B26] Huebner RJ, Todaro GJ (1969). Oncogenes of RNA tumor viruses as determinants of cancer. Proc Natl Acad Sci U S A.

[B27] Weiss RA, Payne LN (1971). The heritable nature of the factor in chicken cells which acts as a helper virus for Rous sarcoma virus. Virology.

[B28] Vogt PK, Friis RR (1971). An avian leukosis virus related to RSV(O): properties and evidence for helper activity. Virology.

[B29] Weiss RA, Friis RR, Katz E, Vogt PK (1971). Induction of avian tumor viruses in normal cells by physical and chemical carcinogens. Virology.

[B30] Astrin SM, Robinson HL, Crittenden LB, Buss EG, Wyban J, Hayward WS (1980). Ten genetic loci in the chicken that contain structural genes for endogenous avian luekosis viruses.

[B31] Baltimore D (1970). RNA-dependent DNA polymerase in virions of RNA tumour viruses. Nature.

[B32] Temin HM, Mizutani S (1970). RNA-dependent DNA polymerase in virions of Rous sarcoma virus. Nature.

[B33] Rosenthal PN, Robinson HL, Robinson WS, Hanafusa T, Hanafusa H (1971). DNA in uninfected and virus-infected cells complementary to avian tumor virus RNA. Proc Natl Acad Sci U S A.

[B34] Varmus HE, Weiss RA, Friis RR, Levinson W, Bishop JM (1972). Detection of avian tumor virus-specific nucleotide sequences in avian cell DNAs. Proc Natl Acad Sci U S A.

[B35] Baluda MA (1972). Widespread presence, in chickens, of DNA complementary to the RNA genome of avian leukosis viruses. Proc Natl Acad Sci U S A.

[B36] Humphries EH, Glover C, Weiss RA, Arrand JR (1979). Differences between the endogenous and exogenous DNA sequences of Rous-associated virus-O. Cell.

[B37] Boeke JD, Stoye JP, Coffin JM, Hughes SH and Varmus HE (1997). Retrotransponsons, endogenous retroviruses and the evolution of retroelements. Retroviruses.

[B38] Weiss RA, Biggs PM (1972). Leukosis and Marek's disease viruses of feral red jungle flow and domestic fowl in Malaya. J Natl Cancer Inst.

[B39] Frisby DP, Weiss RA, Roussel M, Stehelin D (1979). The distribution of endogenous chicken retrovirus sequences in the DNA of galliform birds does not coincide with avian phylogenetic relationships. Cell.

[B40] Weiss RA, Mason WS, Vogt PK (1973). Genetic recombinants and heterozygotes derived from endogenous and exogenous avian RNA tumor viruses. Virology.

[B41] Payne LN, Pani PK, Weiss RA (1971). A dominant epistatic gene which inhibits cellular susceptibility to RSV(RAV-O). J Gen Virol.

[B42] Astrin SM, Buss EG, Haywards WS (1979). Endogenous viral genes are non-essential in the chicken. Nature.

[B43] Boyce-Jacino MT, O'Donoghue K, Faras AJ (1992). Multiple complex families of endogenous retroviruses are highly conserved in the genus Gallus. J Virol.

[B44] Bai J, Payne LN, Skinner MA (1995). HPRS-103 (exogenous avian leukosis virus, subgroup J) has an env gene related to those of endogenous elements EAV-0 and E51 and an E element found previously only in sarcoma viruses. J Virol.

[B45] van der Kuyl AC, Mang R, Dekker JT, Goudsmit J (1997). Complete nucleotide sequence of simian endogenous type D retrovirus with intact genome organization: evidence for ancestry to simian retrovirus and baboon endogenous virus. J Virol.

[B46] Chai N, Bates P (2006). Na+/H+ exchanger type 1 is a receptor for pathogenic subgroup J avian leukosis virus. Proc Natl Acad Sci U S A.

[B47] Isfort RJ, Qian Z, Jones D, Silva RF, Witter R, Kung HJ (1994). Integration of multiple chicken retroviruses into multiple chicken herpesviruses: herpesviral gD as a common target of integration. Virology.

[B48] Hertig C, Coupar BE, Gould AR, Boyle DB (1997). Field and vaccine strains of fowlpox virus carry integrated sequences from the avian retrovirus, reticuloendotheliosis virus. Virology.

[B49] Singh P, Schnitzlein WM, Tripathy DN (2003). Reticuloendotheliosis virus sequences within the genomes of field strains of fowlpox virus display variability. J Virol.

[B50] Rowe WP, Pincus T (1972). Quantitative studies of naturally occurring murine leukemia virus infection of AKR mice. J Exp Med.

[B51] Stoye JP (1998). Fv1, the mouse retrovirus resistance gene. Rev Sci Tech.

[B52] Aaronson SA, Hartley JW, Todaro GJ (1969). Mouse leukemia virus: "spontaneous" release by mouse embryo cells after long-term in vitro cultivation. Proc Natl Acad Sci U S A.

[B53] Lowy DR, Rowe WP, Teich N, Hartley JW (1971). Murine leukemia virus: high-frequency activation in vitro by 5-iododeoxyuridine and 5-bromodeoxyuridine. Science.

[B54] Aaronson SA, Todaro GJ, Scolnick EM (1971). Induction of murine C-type viruses from clonal lines of virus-free BALB-3T3 cells. Science.

[B55] Lieberman M, Kaplan HS (1959). Leukemogenic activity of filtrates from radiation-induced lymphoid tumors of mice. Science.

[B56] Latarjet R, Duplan JF (1962). Experiment and discussion on leukaemogenesis by cell-free extracts of radiation-induced leukaemia in mice. Int J Radiat Biol.

[B57] Rosenberg N, Jolicoeur P, Coffin JM, Hughes SH and Varmus HE (1997). Retroviral Pathogenesis. Retroviruses.

[B58] Benveniste RE, Lieber MM, Livingston DM, Sherr CJ, Todaro GJ, Kalter SS (1974). Infectious C-type virus isolated from a baboon placenta. Nature.

[B59] Benveniste RE, Todaro GJ (1974). Evolution of C-type viral genes: inheritance of exogenously acquired viral genes. Nature.

[B60] Benveniste RE, Heinemann R, Wilson GL, Callahan R, Todaro GJ (1974). Detection of baboon type C viral sequences in various primate tissues by molecular hybridization. J Virol.

[B61] Venables PJ, Brookes SM, Griffiths D, Weiss RA, Boyd MT (1995). Abundance of an endogenous retroviral envelope protein in placental trophoblasts suggests a biological function. Virology.

[B62] Mi S, Lee X, Li X, Veldman GM, Finnerty H, Racie L, LaVallie E, Tang XY, Edouard P, Howes S, Keith JCJ, McCoy JM (2000). Syncytin is a captive retroviral envelope protein involved in human placental morphogenesis. Nature.

[B63] Mallet F, Bouton O, Prudhomme S, Cheynet V, Oriol G, Bonnaud B, Lucotte G, Duret L, Mandrand B (2004). The endogenous retroviral locus ERVWE1 is a bona fide gene involved in hominoid placental physiology. Proc Natl Acad Sci U S A.

[B64] Bentvelzen P, Daams JH (1969). Heredity infections with mammary tumor viruses in mice. J Natl Cancer Inst.

[B65] Bentvelzen P, Daams JH, Hageman P, Calafat J (1970). Genetic transmission of viruses that incite mammary tumor in mice. Proc Natl Acad Sci U S A.

[B66] Cohen JC, Varmus HE (1979). Endogenous mammary tumour virus DNA varies among wild mice and segregates during inbreeding. Nature.

[B67] Acha-Orbea H, Held W, Waanders GA, Shakhov AN, Scarpellino L, Lees RK, MacDonald HR (1993). Exogenous and endogenous mouse mammary tumor virus superantigens. Immunol Rev.

[B68] Levy JA (1973). Xenotropic viruses: murine leukemia viruses associated with NIH Swiss, NZB, and other mouse strains. Science.

[B69] Palmarini M, Mura M, Spencer TE (2004). Endogenous betaretroviruses of sheep: teaching new lessons in retroviral interference and adaptation. J Gen Virol.

[B70] McAllister RM, Nicolson M, Gardner MB, Rongey RW, Rasheed S, Sarma PS, Huebner RJ, Hatanaka M, Oroszlan S, Gilden RV, Kabigting A, Vernon L (1972). C-type virus released from cultured human rhabdomyosarcoma cells. Nat New Biol.

[B71] Achong BG, Trumper PA, Giovanella BC (1976). C-type virus particles in human tumours transplanted into nude mice.. Brit J Cancer.

[B72] Urisman A, Molinaro RJ, Fischer N, Plummer SJ, Casey G, Klein EA, Malathi K, Magi-Galluzzi C, Tubbs RR, Ganem D, Silverman RH, Derisi JL (2006). Identification of a Novel Gammaretrovirus in Prostate Tumors of Patients Homozygous for R462Q RNASEL Variant. PLoS Pathog.

[B73] Patience C, Takeuchi Y, Weiss RA (1997). Infection of human cells by an endogenous retrovirus of pigs.. Nature Med.

[B74] Magre S, Takeuchi Y, Bartosch B (2003). Xenotransplantation and pig endogenous retroviruses. Rev Med Virol.

[B75] Weiss RA (1982). Retroviruses produced by hybridomas. N Engl J Med.

[B76] Purcell DF, Broscius CM, Vanin EF, Buckler CE, Nienhuis AW, Martin MA (1996). An array of murine leukemia virus-related elements is transmitted and expressed in a primate recipient of retroviral gene transfer. J Virol.

[B77] Patience C, Takeuchi Y, Cosset FL, Weiss RA (1998). Packaging of endogenous retroviral sequences in retroviral vectors produced by murine and human packaging cells. J Virol.

[B78] Patience C, Switzer WM, Takeuchi Y, Griffiths DJ, Goward ME, Heneine W, Stoye JP, Weiss RA (2001). Multiple groups of novel retroviral genomes in pigs and related species. J Virol.

[B79] Martin J, Kabat P, Tristem M, Page RDM (2002). Cospeciation and horizontal transmission in the murine leukaemia-related retroviruses. Tangled trees: phylogenies, cospeciation, and coevolution (2003).

[B80] Tarlinton RE, Meers J, Young PR (2006). Retroviral invasion of the koala genome. Nature.

[B81] Teich NM, Weiss RA, Martin GR, Lowy DR (1977). Virus infection of murine teratocarcinoma stem cell lines. Cell.

[B82] Gorman CM, Rigby PW, Lane DP (1985). Negative regulation of viral enhancers in undifferentiated embryonic stem cells. Cell.

[B83] Daibata M, Taguchi T, Nemoto Y, Taguchi H, Miyoshi I (1999). Inheritance of chromosomally integrated human herpesvirus 6 DNA. Blood.

[B84] Tanaka-Taya K, Sashihara J, Kurahashi H, Amo K, Miyagawa H, Kondo K, Okada S, Yamanishi K (2004). Human herpesvirus 6 (HHV-6) is transmitted from parent to child in an integrated form and characterization of cases with chromosomally integrated HHV-6 DNA. J Med Virol.

